# Hereditary cancer registries improve the care of patients with a genetic predisposition to cancer: contributions from the Dutch Lynch syndrome registry

**DOI:** 10.1007/s10689-016-9897-1

**Published:** 2016-03-14

**Authors:** Hans F. A. Vasen, Mary E. Velthuizen, Jan H. Kleibeuker, Fred H. Menko, Fokke M. Nagengast, Annemieke Cats, Andrea E. van der Meulen-de Jong, Martijn H. Breuning, Anne J. Roukema, Inge van Leeuwen-Cornelisse, Wouter H. de Vos tot Nederveen Cappel, Juul T. Wijnen

**Affiliations:** Department of Gastroenterology and Hepatology, Leiden University Medical Centre, Albinusdreef 2, 2333 ZA Leiden, The Netherlands; Hereditary Cancer Registry, Leiden, The Netherlands; Department of Clinical Genetics, University Medical Centre, Utrecht, The Netherlands; Department of Gastroenterology and Hepatology, University Medical Centre Groningen, Groningen, The Netherlands; Cancer Family Clinic, Netherlands Cancer Institute, Amsterdam, The Netherlands; Department of Gastroenterology and Hepatology, Slingerland Ziekenhuis, Doetinchem, The Netherlands; Department of Gastroenterology and Hepatology, Netherlands Cancer Institute, Amsterdam, The Netherlands; Department of Clinical Genetics, Leiden University Medical Centre, Leiden, The Netherlands; Department of Surgery, Elizabeth Hospital, Tilburg, The Netherlands; Department of Gastroenterology and Hepatology, Isala Clinics, Zwolle, The Netherlands

**Keywords:** Hereditary cancer, Registry, Follow-up system, Identification, Lynch syndrome, Cancer risk, Surveillance

## Abstract

The Dutch Hereditary Cancer Registry was established in 1985 with the support of the Ministry of Health (VWS). The aims of the registry are: (1) to promote the identification of families with hereditary cancer, (2) to encourage the participation in surveillance programs of individuals at high risk, (3) to ensure the continuity of lifelong surveillance examinations, and (4) to promote research, in particular the improvement of surveillance protocols. During its early days the registry provided assistance with family investigations and the collection of medical data, and recommended surveillance when a family fulfilled specific diagnostic criteria. Since 2000 the registry has focused on family follow-up, and ensuring the quality of surveillance programs and appropriate clinical management. Since its founding, the registry has identified over 10,000 high-risk individuals with a diverse array of hereditary cancer syndromes. All were encouraged to participate in prevention programmes. The registry has published a number of studies that evaluated the outcome of surveillance protocols for colorectal cancer (CRC) in Lynch syndrome, as well as in familial colorectal cancer. In 2006, evaluation of the effect of registration and colonoscopic surveillance on the mortality rate associated with colorectal cancer (CRC) showed that the policy led to a substantial decrease in the mortality rate associated with CRC. Following discovery of MMR gene defects, the first predictive model that could select families for genetic testing was published by the Leiden group. In addition, over the years the registry has produced many cancer risk studies that have helped to develop appropriate surveillance protocols. Hereditary cancer registries in general, and the Lynch syndrome registry in particular, play an important role in improving the clinical management of affected families.

## Introduction

The Dutch Hereditary Cancer Registry was established in 1985 [1, 2]. Up to 2013 the registry was financed by the Ministry of Health (VWS), but it is now being financed by Dutch hospitals and insurance companies. The aims of the registry are: (1) to promote the identification of families with hereditary cancer, (2) to encourage high-risk individuals to participate in surveillance programs, (3) to ensure the continuity of the surveillance examinations which are required lifelong, and (4) to promote research, in particular the improvement of surveillance protocols.

**Fig. 1:**
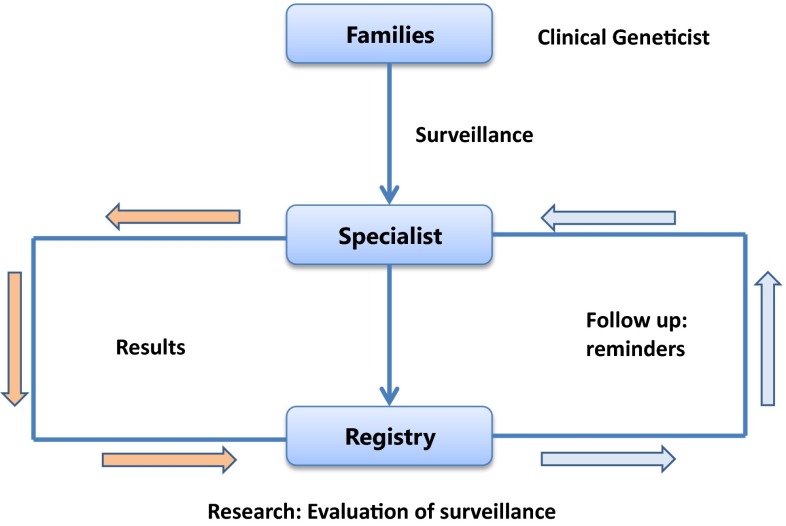
Methodology of the registry 1985–2015

The approach developed by the registry was simple but wide-ranging: we first established collaborations with all major gastroenterology departments in the Netherlands, and then formed a national multidisciplinary collaborative group that consisted of physicians with an interest in hereditary CRC. During the early years, data were collected locally at each collaborating institution on previously identified families (in particular, polyposis and Lynch syndrome families) and family investigations were also offered. When dealing with very large families, we organised local meetings (similar to the Family Information Service (FIS) methods described by Lynch [3]) in order to inform family members about the syndromes and about surveillance options. In the 1990’s, following the discovery of the major gene defects responsible for most of the hereditary cancer syndromes, family cancer clinics were established all over the country and proceeded to offer presymptomatic testing. At that time we opened discussions with the Dutch Association of Clinical Genetic Centres on how tasks could be distributed between the registry and family cancer clinics. It was agreed that the clinical genetic centres would take responsibility for family investigations, genetic counselling, genetic testing and provision of up-to-date information on screening programs. The task of the registry would be to focus on follow-up of the families over their lifetime, and on the quality of surveillance programs and clinical management. This approach is schematically illustrated in Fig. 1. Since 2000, clinical geneticists refer families with a proven mutation to both the registry and the clinical specialist (e.g., gastroenterologist, gynaecologist) for surveillance. The results of screening by the clinical specialist are shared with the registry, and at regular intervals (1–3 years depending on the disorder) the registry sends out surveillance reminders to the specialists. To date, the registry has identified over 10,000 high-risk individuals with various hereditary cancer syndromes (Table 1), all of whom were encouraged to participate in prevention programmes.

**Table 1:** Number of registered individuals per hereditary tumour syndrome (2014)

Disorder	N
Familial adenomatous polyposis	3700
HNPCC/Lynch Syndrome	3020
Familial CRC	550
Hereditary breast (ovarian) cancer	2700
FAMMM	2570
Hereditary prostate cancer	1005

In 2006 we carried out an evaluation of the effect of registration, followed by surveillance. At that time 140 families with Lynch syndrome were registered, including nearly 3000 mutation carriers and their first-degree relatives. The standard mortality rate (SMR) associated with CRC (the mortality rate associated with CRC observed in the families relative to the mortality rate of CRC in the general population) was calculated for three periods of 15 years, and it was found that registration together with surveillance led to a substantial decrease in the SMR [4].

A meta-analysis of the effect of registration and screening on the CRC mortality rate in both Lynch syndrome and familial adenomatous polyposis (FAP) was recently performed by Barrow et al. [5.] The results regarding Lynch syndrome confirmed our findings.

The initial success of the registry was mainly due to the large numbers of families that were rapidly identified by our highly-motivated genetic counsellors and registry administrative staff. Next, we established successful national and international collaborations. International collaboration started with the launch of the International Collaborative Group on HNPCC (ICG-HNPCC), its first meeting organised by the registry in Amsterdam in 1990 [6]. All subsequent meetings over the first 10 years of the collaboration were organised by the Dutch registry, together with local organisers. In 2006 a European collaborative group was established by the registry, together with our German colleague (Gabriela Moslein).

The current chapter will address three questions: (1) how can we identify families at risk for Lynch syndrome, (2) what are the risks of developing CRC and other cancers, and (3) how effective are the screening programs for CRC and other cancers. In particular, we discuss the contributions of the Dutch registry to resolving these issues.

### Identification

At the time of founding of the registry in 1985, Lynch syndrome (LS) was still a relatively little-known disorder and only a few families had been described in the literature, mainly by Henry T. Lynch. When establishing a new syndrome several criteria need to be met, including [1] an appropriate name, [2] clinical diagnostic criteria, and [3] ideally a known underlying genetic defect. When a gene is identified it is important to determine which families should be tested [4]. Finally, families that should be tested using specific markers (MSI, see below) need to be identified [5].

In the 1980s, Lynch syndrome was referred to by many different names such as ‘cancer family syndrome’, ‘hereditary site-specific CRC’, ‘Lynch Syndrome type 1’, ‘Lynch Syndrome type 2’, etc. [7] One of the first achievements of the ICG-HNPCC was the proposal of a uniform term for Lynch syndrome, ‘hereditary non-polyposis colorectal cancer’ (HNPCC) [6]. At the time this name was particularly useful in clinical practice because it specified a hereditary form of CRC that could then be better differentiated from familial adenomatous polyposis (FAP), already a well-known syndrome by the 1980s. The group also proposed clinical diagnostic criteria, the well-known Amsterdam criteria, which were particularly useful in identifying families suitable for research [6, 8].

During the 1990s, the molecular basis of Lynch syndrome and all major underlying gene defects were discovered within a very short period [9]. The high costs of mutation analysis at the time meant that it was important to select the appropriate families for genetic testing. Based on the outcome of genetic testing of families known to the registry, the Leiden group developed the first predictive model that could be used for this purpose [10]. Nowadays, several widely-used models are available [11].

In 1993, a Finnish study reported that tumours associated with LS were characterised by the presence of microsatellite instability (MSI) [12]. This important observation provided a new means of identifying families with LS. Subsequently, (the Bethesda) guidelines for MSI were developed based on the cardinal features of LS: early age of onset of CRC, multiple tumours in one individual and multiple family members with a tumour [13]. A major problem was that a detailed family history was required to evaluate whether a family complied with the Amsterdam criteria or Bethesda guidelines. However, numerous studies have shown that family history is often neglected in clinical practice [14]. A new approach that solved this problem was suggested by Hampel and co-workers—the screening of all new CRC cases by MSI analysis (or IHC) [15]. They performed MSI analysis (and immunohistochemical MMR protein analysis) followed by mutation analysis of the positive cases in a large series of unselected CRC cases. A mutation was identified in about 2.5 % of all CRC cases, in addition to a large number of relatives of index patients who were found to carry a predisposing mutation.

Based on these findings the authors recommended screening of all new CRCs for MSI or IHC, independent of the family history. Subsequent studies confirmed these findings and reported a detection rate of 3–4 % for MMR gene defects in unselected CRC cases [16]. Moreover, various studies showed that this approach was cost-effective [17], and universal screening has now been implemented in many countries.

### Cancer risk in Lynch syndrome

Over the years, the Dutch registry has contributed many studies on the risk of developing cancer [18–26]. The first of these described the tumour spectrum associated with Lynch syndrome [22]. The Dutch registry also published the first estimates for cancer risk in carriers with a proven MMR gene defect [23]. In collaboration with the Omaha registry, data were published on the risk of endometrial cancer and extra-colonic cancer [27, 28]. The registry also collaborated with the German HNPCC Consortium and reported risk for extracolonic cancers evaluated in 2118 carriers, the largest series of carriers to date [29].

The largest current study on cancer risk in MSH6 carriers was reported by Baglietto et al. [30], a study that included a large proportion of families contributed by the Dutch registry. In 2015 the Leiden group, in collaboration with the Dutch registry, described cancer risks in a large series of PMS2 mutation carriers [31].

In Table 2, a summary is shown of the CRC and EC risk estimates for the various gene defects [30–32]. The age of onset of CRC and EC is delayed by about 5–10 years in carriers of MSH6 or PMS2 mutations compared to carriers of MLH1 or MSH2 mutations. Moreover, the risk of CRC and EC is substantially lower in the carriers of an MSH6 or PMS2 mutation compared to risk in carriers of other MMR defects.

**Table 2:** Cancer risk in carriers of various MMR gene defects

	MLH1/MSH2	MSH6	PMS2
*CRC*			
Mean age (years)	45	56	52
Risk 70 years (M/F)	53/33 %	22/10 %	19/11 %

	MLH1/MSH2*	MSH6	PMS2
*EC*			
Mean age (years)	45	52	55
Risk 70 years (F)	44 %	26 %	12 %

* EPCAM 12 %

Risk estimates for extra-colonic cancer development are summarized in Table 3 [16, 29–35]. The data show that MSH6 mutation carriers have the lowest risk for the non-CRC and non-endometrial cancers. MSH2 and MLH1 mutation carriers have the highest risk estimates. Information in the literature on cancer risk for carriers of a PMS2 mutation is limited and only estimates of relative risk (RR) are available.

**Table 3:** Summary of extra-colonic cancer risk for respective gene defect

	MLH1* (%)	MSH2* (%)	MSH6* (%)	PMS2
Urinary tract	1–3	8–10	0–1	RR renal pelvis: 50
Ovary	11	10	1	RR 12
Gastric	8	2–9	<4	RR 0
Small bowel	5	3	0	RR 115
Prostate	–	17	–	RR 1.7
Biliary/pancreatic	3	4	0	–
Brain	1	4	0	RR 2.7

* Life time risk by age 70

In conclusion, diverse studies have consistently shown a substantial difference in cancer risk associated with the various gene defects. Consequently, carriers of an MMR defect require tailored management depending of the underlying gene defect. To ensure that these carriers receive appropriate management, it has been suggested that the underlying gene defect should be included in the name of the syndrome (e.g. MLH1-Lynch syndrome) [36].

### Lynch syndrome surveillance

In 1990, the ICG-HNPPC recommended a surveillance interval of 2–3 years between colonoscopies. Five years later, the Dutch registry identified six patients who developed an (interval) cancer within 2–3 years of a normal colonoscopy [37]. During the same period, studies by Henry Lynch, Steven Lanspa and Jeremy Jass also indicated that carcinogenesis (adenoma-carcinoma sequence) in Lynch syndrome was accelerated [38, 39]. This insight has since led to a shorter screening interval and 1–2 years is now recommended.

In 2010, the effectiveness of this approach was evaluated using data from the Dutch registry. It was found that the risk of developing an interval cancer was relatively low: 6 % after 10 years of follow-up [40]. Moreover, most screen-detected tumours were early (stage 1 and 2) cancers. The type of gene defect and the current age of the high-risk individual were found to influence the risk of developing an interval CRC. An MSH6 mutation and age <40 years was associated with a (albeit non-significant) lower risk. Based on these findings, up to age 40 a 2-year interval can be recommended for carriers of an MLH1 or MSH2 mutation, with a more intensive program (interval 1–2 years) from age 40 years. In carriers of an MSH6 mutation or PMS2 mutation, an intensive protocol (1–2 years) may be recommended after age 50. A recent study, presented at the InSiGHT meeting 2015 in Sao Paulo, evaluated the effect of the length of the screening interval on survival and the cumulative incidence of CRC. The investigators collected data on almost 1000 carriers of an MLH1 mutation, half of which were from Finland where a 2–3 year interval is advised, with the other half collected from various European countries where 1–2 years between examinations is the generally recommended interval. The results showed that survival was improved in individuals who developed an interval cancer (although non-significant) during the shorter (1–2 year) interval. However, one interesting finding was that cumulative CRC incidence was significantly lower in Finland (despite the 2–3 year interval) compared to other countries. One explanation might be that the overall risk of developing CRC in the general population is substantially lower in Finland compared to other countries.

A summary of the prevention program for Lynch syndrome patients is shown in Table 4. Unfortunately, the value of surveillance for most cancers (e.g., endometrium, urinary tract, gastric cancer, small bowel cancer) associated with Lynch syndrome is unknown.

**Table 4:** Prevention program in Lynch Syndrome

Surveillance
MLH1: CRC, EC/OC
MSH2: CRC, EC/OC, urinary tract
MSH6: CRC, EC/OC
PMS2: CRC, EC/OC
Assessment of H. Pylori (biopsy, faeces, serology)
Discuss options for prophylactic surgery (uterus, ovaries)
General recommendations: no smoking, BMI < 25

*CRC* colorectal cancer, *EC* endometrial cancer, *OC* ovarian cancer

Most guidelines therefore recommend screening for these particular cancers within a research setting or in those individuals whose screening results are collected by a hereditary cancer registry.

An example of this type of study is a recent report on the value of videocapsule endoscopy for the early detection of small bowel cancer. In this study a total of 200 mutation carriers underwent videocapsule endoscopy (VCE), which resulted in the detection of only one duodenal cancer and one duodenal adenoma [41]. One patient developed a duodenal cancer 7 months after a negative VCE. Regrettably, the outcome does not allow conclusions to be drawn on the value of VCE in the surveillance of small bowel cancer.

There is general agreement that the option of prophylactic surgery (hysterectomy and bilateral salpingo-oophorectomy) for mutation carriers from age 40 and with a complete family history should be discussed, dependent on the underlying gene defect. Finally, recommendations on lifestyle factors and nutrition (avoid smoking, keep BMI below 25) should be provided [42–44].

### Surveillance of familial colorectal cancer

An important question is which surveillance program should be recommended for families with clustering of CRC but without evidence of MMR deficiency. Familial CRC may be subdivided into three categories: (1) familial colorectal cancer syndrome type X—these include families that comply with the Amsterdam criteria but without evidence of MMR deficiency [45], (2) late-onset familial clustering of colorectal cancer—families similar to [1] but all CRC cases diagnosed above the age of 50 years [46], and (3) familial CRC ss (sensu strictu)—families with one first-degree relative with CRC <50 years or two first-degree relatives with CRC.

A team of investigators from St Mark’s hospital evaluated the effectiveness of surveillance (3–5 year interval) for the first two categories of familial CRC in a large series (approx. 1000) of high-risk family members [47]. They showed that the risk of developing CRC under surveillance was low (<5 %) and the risk for high-risk adenoma was also relatively low (<20 %).

In the Netherlands, a randomized controlled trial in familial colorectal cancer (category 3) was recently completed [48]. The aims were: (1) to assess the appropriate surveillance interval, and (2) to identify risk factors for the development of AAP at follow-up. A total of 550 individuals at risk for familial colorectal cancer participated in the study. The participants were subdivided according to the findings at the baseline colonoscopy. Patients with 0–2 adenomas were randomized into two groups, A and B. Group A underwent colonoscopy after 6 years and group B at 3 and 6 years. The endpoint of the study was the presence of an advanced adenomatous polyp (AAP). The results showed that the frequency of AAP at 6-year follow-up was two-fold higher (but not significant) compared to the frequency after 3 years. The presence of AAP at baseline was found to be a significant predictive factor for the development of AAP at follow-up. After correction for AAP at baseline, the difference in the frequency of AAP between group A and group B was significant. However, the absolute risk of developing AAP after 6 years was relatively low (6.9 %). Moreover, none of the participants in group A instead of B developed CRC. Therefore, we consider an interval of 6 years to be safe. However, when an AAP is detected at baseline the colonoscopy should be repeated after 3 years.

## Conclusion

The studies conducted by the Dutch registry have contributed substantially to the understanding and appropriate care of Lynch syndrome patients. A major step forward in the identification of Lynch syndrome families has been the use of universal screening of all new cases of CRC and EC < age 70 by MSI analysis or immunohistochemical analysis of the MMR proteins; hopefully this approach will eventually allow the detection of all Lynch syndrome cases. In the coming years, the use of universal screening should be evaluated for other cancers associated with Lynch syndrome (ovarian cancers, small bowel cancer, gastric cancer, urinary tract cancer, and sebaceous tumours).

All studies that have evaluated cancer risk in Lynch syndrome patients have shown substantial differences in risk between carriers of MLH1 or MSH2 mutations and those with MSH6 or PMS2 mutations. This has important implications for the surveillance protocols used in these families. In order to facilitate personalized management, we propose that the gene defect is included with the name of the syndrome (e.g., MSH2-Lynch syndrome, etc.) [36].

The currently recommended surveillance interval (1–2 years) for Lynch syndrome families appears to be safe. Future studies should evaluate the value of surveillance of other LS-associated cancers. Because there is no evidence of accelerated carcinogenesis in families with familial colorectal cancer, an interval of 3–6 years is generally advised.

The establishment of the ICG-HNPCC had led to worldwide collaboration in the field of Lynch syndrome. Henry Lynch, the first chairman of the group, was the motor behind all these activities. He has been a constant inspiration, an example and a friend to all researchers and healthcare providers in the field.
